# InGaN blue resonant cavity micro-LED with RGY quantum dot layer for broad gamut, efficient displays

**DOI:** 10.1186/s11671-024-04018-4

**Published:** 2024-04-30

**Authors:** Tzu-Yi Lee, Chien-Chi Huang, Yu-Ying Hung, Fang-Chung Chen, Yu-Heng Hong, Hao-Chung Kuo

**Affiliations:** 1 Department of Photonics, College of Electrical and Computer Engineering, National Yang Ming Chiao Tung University, Hsinchu, 30010 Taiwan; 2 Semiconductor Research Center, Hon Hai Research Institute, Taipei, 11492 Taiwan

**Keywords:** Quantum dot, Micro resonant cavity light emitting diode, Color conversion layer, Four-color subpixels, Atomic layer deposition

## Abstract

The technology of RGBY micro resonant cavity light emitting diodes (micro-RCLEDs) based on quantum dots (QDs) is considered one of the most promising approaches for full-color displays. In this work, we propose a novel structure combining a high color conversion efficiency (CCE) QD photoresist (QDPR) color conversion layer (CCL) with blue light micro RCLEDs, incorporating an ultra-thin yellow color filter. The additional TiO_2_ particles inside the QDPR CCL can scatter light and disperse QDs, thus reducing the self-aggregation phenomenon and enhancing the eventual illumination uniformity. Considering the blue light leakage, the influences of adding different color filters are investigated by illumination design software. Finally, the introduction of low-temperature atomic layer deposition (ALD) passivation protection technology at the top of the CCL can enhance the device's reliability. The introduction of RGBY four-color subpixels provides a viable path for developing low-energy consumption, high uniformity, and efficient color conversion displays.

## Introduction

As the global display market continually seeks higher image quality, enhanced color accuracy, reduced power consumption, and wider color gamuts and application scopes, resonant cavity light emitting diode (RCLED) technology emerges as a specially designed LED within the modern full-color display technology domain [1, 2]. Consisting of distributed Bragg reflectors (DBR) at both ends, it forms an optical resonant cavity that enhances the intensity of light at specific wavelengths through multiple reflections within the cavity, thereby improving the light's output efficiency and directionality [3, 4]. Moreover, by adjusting the length of the resonant cavity, the wavelength of the light can be precisely controlled, allowing for exact color emission. Compared to traditional micro-LEDs, RCLEDs excel in color and energy efficiency performance. The resonant cavity structure of RCLED technology enhances the light's output efficiency and color purity, resulting in more vivid and accurate colors, significantly elevating the quality of full-color displays [4, 5]. This technology is particularly suited for applications requiring high color accuracy and wide color gamuts, such as professional-grade displays and high-end consumer electronics. Although traditional micro-LED technology performs well in brightness and energy efficiency, it may not reach the high standards of color purity and consistency offered by RCLEDs [2, 6].

In display technology, traditional pixel configurations typically use red, green, and blue (RGB) primaries to produce a wide range of colors [7, 8]. This configuration relies on the theory of color mixing, where different proportions of RGB primary colors are blended to create millions of distinct colors. In recent years, some display technologies have begun to adopt red, green, blue, and yellow (RGBY) pixel configurations, aiming to provide a broader color gamut and improved display effects [9, 10]. Adding yellow pixels can enhance the accuracy and naturalness of certain color representations, such as skin tones, skies, and natural landscapes, making images more vivid and realistic [11]. Due to the high light output efficiency of RCLEDs at specific wavelengths, combining yellow pixels can increase the overall brightness of the display without raising energy consumption, especially notable in presenting bright scenes.

Quantum dot (QD) color conversion technology plays a pivotal role in today's display industry due to its excellent absorption of blue light and narrower full width at half maximum (FWHM). Besides achieving higher color conversion efficiency, it also effectively enhances color purity, yielding a broader color gamut area, making it highly suitable as a color conversion layer (CCL) [12–17]. Combining blue RCLED with red, green, and yellow CCLs achieves high directionality and precise wavelength control, helping to reduce optical crosstalk between different color pixels, thus enhancing color purity and display clarity. However, as display devices continue to become thinner and smaller, QD CCLs, when insufficient in thickness, tend to leak a significant amount of blue light, leading to a substantial drop in light conversion efficiency (LCE) [17]. To address this issue, embedding QDs in nanoporous micro-LEDs can accommodate QDs [18–20] and increase light scattering [13, 21–23], improving the issue of blue light leakage. Additionally, adding nano-sized scattering particles to extend the light path is an excellent solution for enhancing LCE and light intensity. To further reduce blue light leakage, a color filter (CF) can be added on top of the QD CCL, replacing the commonly used DBR, as the production cost and required thickness of DBR are relatively high [24]. Replacing it with a CF can reduce module thickness, benefiting applications in slim or wearable devices. With these enhancements in display performance, we propose a novel full-color display module design utilizing blue RCLED combined with RYG QD CCL, incorporating a four-color full-color display array. Notably, we add scattering particles TiO_2_ and a CF in the QD CCL to minimize blue light leakage. Through our design, we have developed a display module with high efficiency, wide color gamut, and high color accuracy. This innovative combination of technologies not only advances the development of display technology but also promises to deliver a superior visual experience to consumers in the high-end market domain in the future.

## Experiment and fabrication process

In this study, we have successfully developed a full-color micro-RCLED array with high-efficiency quantum dot conversion. The fabrication process of the blue light micro-RCLED array follows the procedure we previously published [25]. Initially, we fabricated blue light micro-RCLEDs with dimensions of 5 × 5 μm and arranged them into an 8 × 8 array. The quantum dot photoresist (QDPR) was prepared by dispersing QDs in the photoresist, with the addition of a quantified amount of nano-particles TiO_2_ (QD:TiO_2_ = 30:17.5), ensuring thorough and uniform mixing. Subsequently, we fabricated the QD CCL array. First, a 2 μm thick layer of black photoresist was deposited on a 150 μm thick high-transparency glass substrate. The black matrix (BM) was used to planarize the micro-RCLED array and prevent lateral leakage of blue light. Next, through photolithography, red QDPR, green QDPR, yellow QDPR, and transparent PR were sequentially prepared to form color pixels (Thickness = 2 μm). The color pixels were designed with dimensions of 5 μm × 5 μm and a spacing of 1 μm between each pixel. Then, a 0.6 μm thick layer of yellow dye was deposited on the pixel surface as a filter. Using low-temperature atomic layer deposition (ALD) technology (50 °C), aluminum oxide (Al_2_O_3_) was deposited on the QDPR array to passivate the QD surface. This passivation layer not only protects the QDs from any changes due to high temperature and material properties but also shields them from moisture and oxidation. Finally, the glass with the color pixel array was bonded to the blue light micro-RCLED array using an aligner and UV resin. This completes the fabrication of the full-color micro-RCLED array.

## Result and discussion

**Fig. 1:**
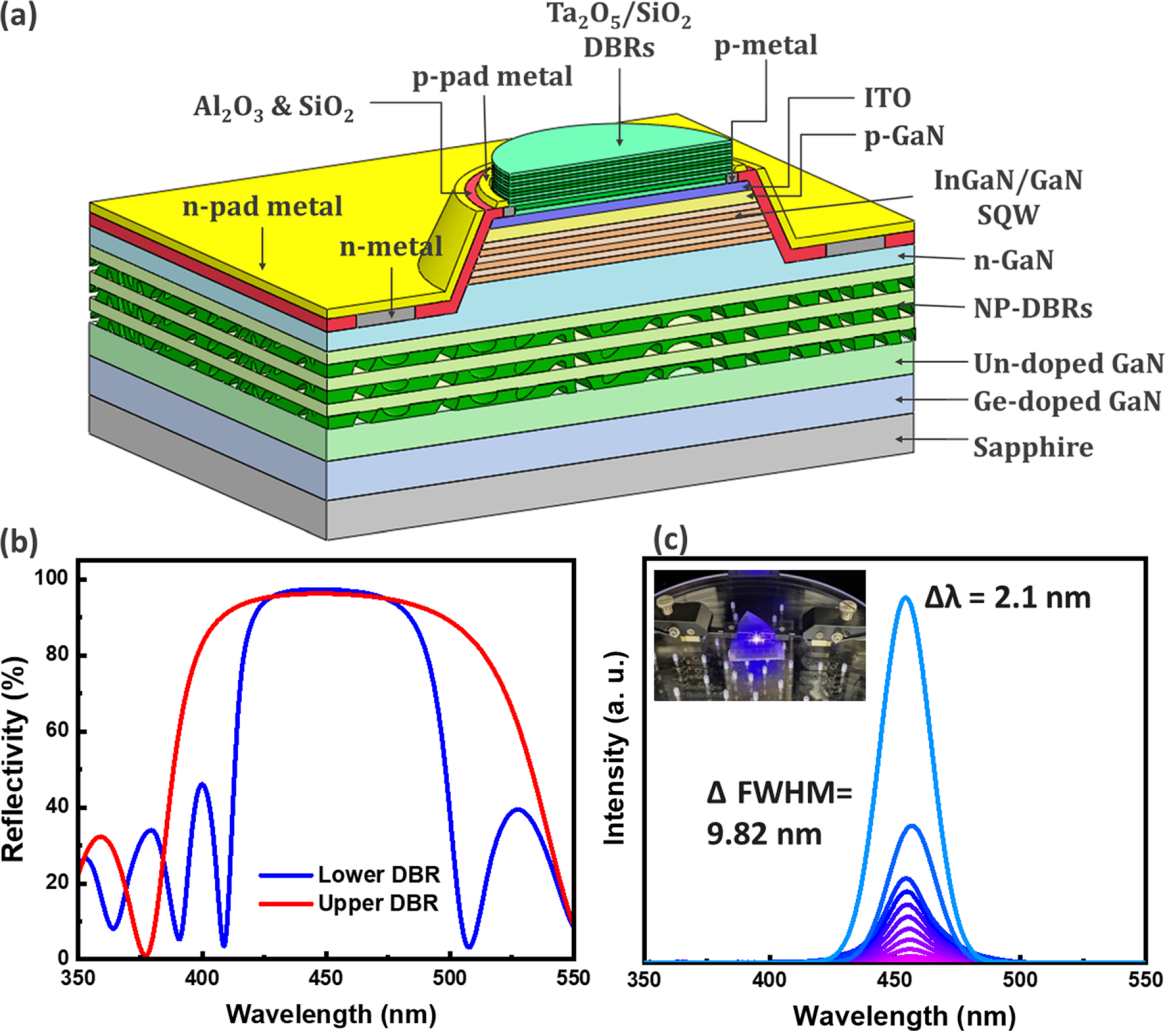
**a** Schematic of blue micro-RCLED **b** Simulated spectra of upper and lower nanoporous (NP)-DBR **c** Electroluminescence spectrum

In this study, we present the structural schematic of a 5 µm blue micro-RCLED array with staggered quantum wells (QWs) grown on a 2-inch polar c-plane (0001) GaN epitaxial wafer, as shown in Fig. 1a. Through bandgap engineering with staggered QWs arrangements, the overlap of wave functions can be enhanced, thereby achieving improved efficiency and wavelength stability for micro-LEDs. Figure 1b shows the reflectance spectrum of the structure’s DBR (NP-DBR) and the top DBR (Ta_2_O_5_/SiO_2_ DBR). The refractive indices of GaN and NP-GaN are approximately 2.39 and 1.7, respectively. The upper and lower DBRs together form a Fabry–Perot cavity, used for filtering the wavelength of emitted light [3, 4, 25]. After the device fabrication, optical properties were measured with a spectrometer (Maya 2000 Pro), where the emitted light was collected by an integrating sphere. Figure 1c displays the electroluminescence (EL) emission spectrum, showing that increasing the input current from 0.17 A/cm^2^ to 2.55 A/cm^2^ led to a peak wavelength shift of about 2.1 nm. This shift places it within the blue spectrum region, belonging to the blue light spectrum. In terms of FWHM, it expanded by 9.82 nm. These results confirm that the blue micro-RCLEDs we prepared exhibit excellent wavelength stability, showing smaller wavelength shifts and narrower FWHM. Compared with the commercially available blue micro LEDs, the blue micro-RCLEDs we developed are more competitive, and some of the data are referenced from our previous study [25]. Both characteristics are advantageous for subsequent applications in full-color displays.

**Fig. 2:**
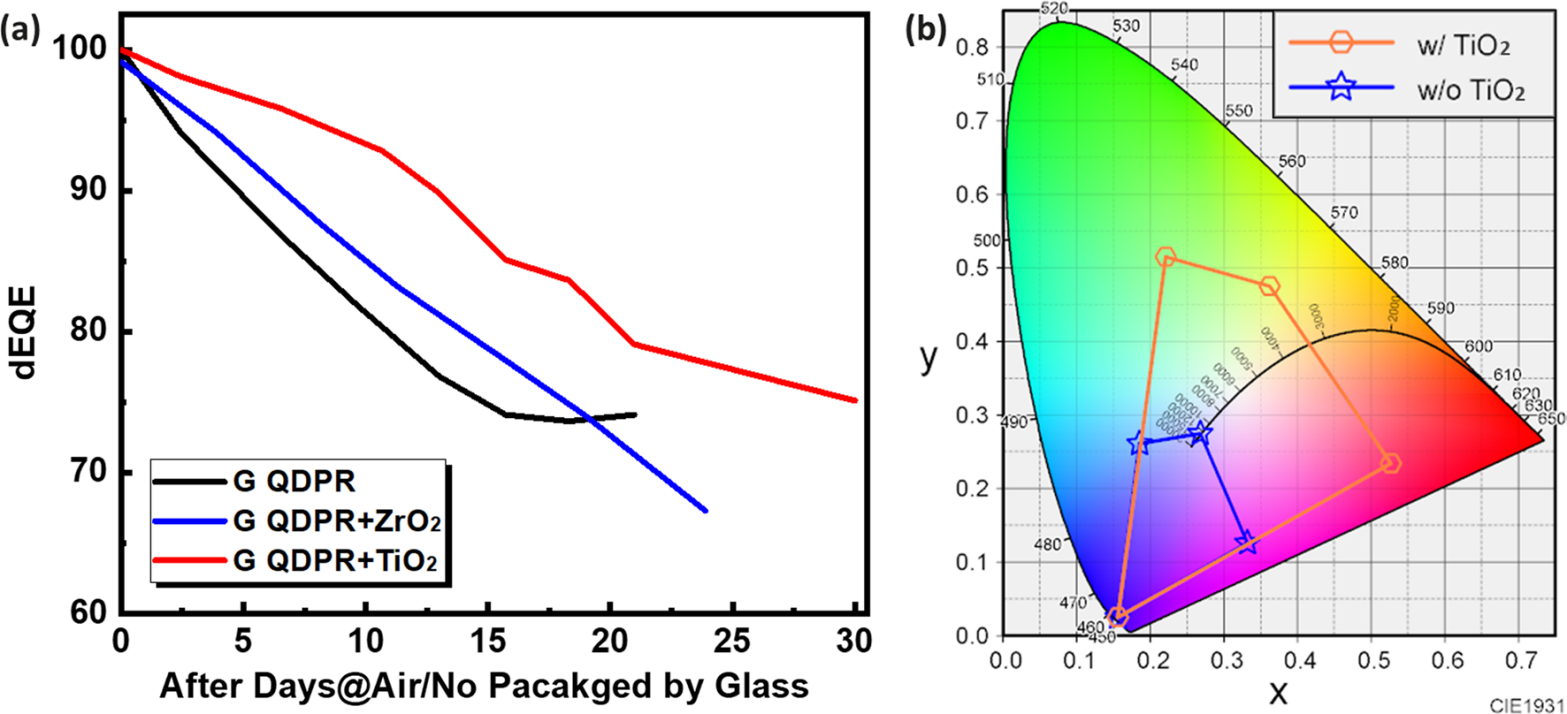
**a** Comparison of reliability performance of QDPR without scattering particles, with ZrO_2_ and with TiO_2_. **b** Comparison of color gamut with and without TiO_2_ scattering particles

In the field of QDs color conversion technology, the incorporation of nanoscale scattering particles significantly enhances light conversion efficiency and display performance. These minute particles scatter incident light, extending the light's path within the quantum dot layer. This extension increases light absorption and conversion efficiency. Furthermore, nano-scattering particles contribute to the uniformity of the light source and enhance the purity and saturation of colors. This improvement is crucial for the development of high-performance displays and optical devices. Commonly used nano-scattering particles include titanium dioxide (TiO_2_)[13, 22], zirconium dioxide (ZrO_2_)[26, 27], silica (SiO_2_)[23], and aluminum oxide (Al_2_O_3_)[28], among others. These particles are selected for their high refractive index, chemical stability, and favorable optical properties, which effectively scatter visible light and boost the luminous efficiency of QDs. They are extensively utilized to enhance the CCL of QDs, providing more uniform light output and improved color performance. The careful selection and application of these nano-scattering particles, based on their specific physical and chemical characteristics, are pivotal in various QD color conversion systems. By precisely engineering the size, shape, and distribution of the particles, the optical performance of the QD CCL can be optimized further. This optimization leads to a more efficient and stable photoelectric conversion effect. In previous research, we explored the advantages of adding TiO_2_ scattering particles in QDPR using simulation software and confirmed that the addition of an appropriate amount of scattering particles can help improve the LCE. [13, 21–23] In this study, we specifically delve into the reliability aspect. Figure 2a compares green QDPR (G QDPR) thin films without scattering particles, with ZrO_2_ added, and with TiO_2_ added, respectively. The testing conditions were set at 25 °C and 50% humidity, and all samples were left unsealed and stored for 30 days for the decrease in EQE (dEQE). The measurement results showed that the QDPR with added TiO_2_ demonstrated superior reliability. This could be due to TiO_2_ having a higher refractive index compared to ZrO_2_, which may offer better performance in enhancing the optical properties of the QD thin films. This optimized optical performance could, to some extent, offset any minor stability issues that might arise during long-term storage. Moreover, incorporating scattering particles into the QDPR is necessary. Figure 2b shows a comparison of the CIE chromaticity coordinates with and without TiO_2_ scattering particles added to the QDPR. After the addition of TiO_2_, the color gamut significantly increases, with an approximate 5 times enhancement, due to improved conversion efficiency. This increase is because the scattering particles extend the light path, boosting the conversion efficiency, and thereby expanding the color gamut area [13].

**Fig. 3:**
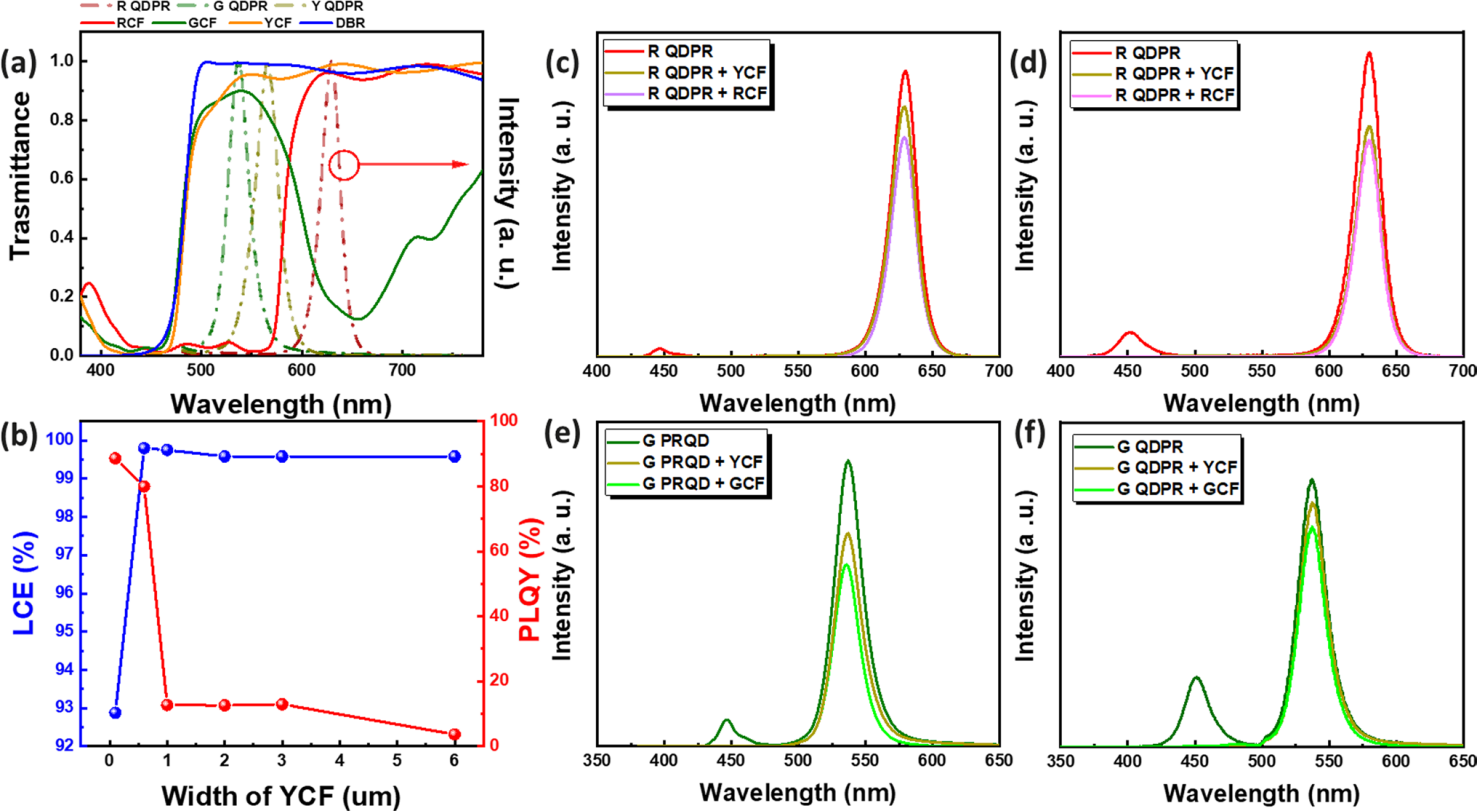
**a** RGY QD emission spectrum with RGY and DBR transmission spectra, comparison of red QDPR without CF with yellow CF or red CF **b** impact of Yellow CF on Efficiency **c** simulation **d** measurement data, green QDPR without CF with yellow CF or red CF **e** simulation **f** measurement data

To further reduce the blue light leakage phenomenon in QD CCLs, this research explores the best color choice for a CF to be added to the surface of QD CCLs. It also compares the differences in LCE brought about by the introduction of DBR, as depicted in Fig. 3. Figure 3a displays the simulated emission spectrum of RGY QDPR along with the transmission spectra of RGY filters and DBR using LightTools (8.6) illumination design software. The thickness of the filter is 0.6 μm, and the DBR is 6 μm thick. From the figure, it is observed that DBR provides the best transmission for RGY and can effectively filter blue light, with yellow CF being the next best option. Green CF shows poorer transmission for the red light spectrum, and red CF has very low transmission for green and yellow light bands. To reduce manufacturing costs and time, yellow CF is the preferred choice. Given the higher production cost and greater thickness of DBR, which is disadvantageous for use in small-sized devices, the yellow CF, with its relatively better performance, was chosen. To determine if a CF thickness of 0.6 µm is optimal, we conducted simulations, as illustrated in Fig. 3b. We varied the yellow CF thickness across 0.1, 0.6, 1, 2, 3, and 6 µm in our simulation framework. The results indicate that at 0.6 µm thickness, the LCE peaks at 99.8%, and the Photoluminescence Quantum Yield (PLQY) reaches 80.0%. While the LCE remains nearly constant with increasing thickness, effectively mitigating blue light leakage, it also diminishes the emission peak intensity. Consequently, the PLQY significantly drops, leading to reduced efficiency. Thus, a CF thickness of 0.6 µm proves to be the most effective choice. Figure 3c–f present the simulated and actual measured spectra of RG QDPR using RGY CF. It is observed that, compared to samples without CF, the addition of CF, although reducing emission intensity, is very effective in suppressing blue light leakage. The difference in emission intensity between using yellow CF and RG CF is not significant. Therefore, yellow CF was ultimately selected as the blue light filtering layer for QD CCLs.

**Fig. 4:**
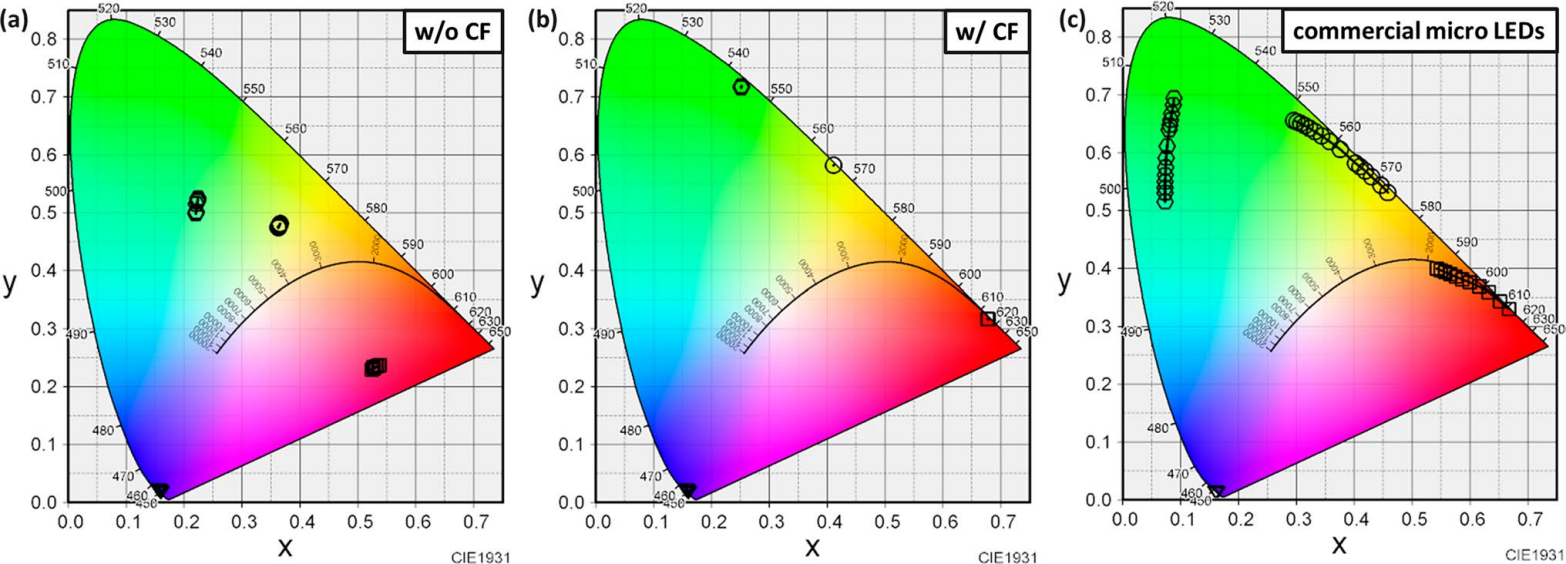
Comparison of the degree of wavelength shift that changes the output power of **a** without CF **b** with CF **c** commercial micro-LED

While blue light micro-RCLEDs have already demonstrated excellent wavelength stability, for high-end display products pursuing high color purity, it is crucial to minimize any slight differences. Fortunately, the emission wavelength of QDs is very stable and does not vary with minor differences in the wavelength of the provided light source. However, leakage of the excitation light source can cause a shift in chromaticity, and it is essential to avoid this phenomenon. We used LightTools (8.6) to simulate the wavelength shift situation of blue light micro-RCLEDs with added QDPR when changing the output power (0.25 ~ 3.5 W). Figure 4 compares the impact of adding a CF on the chromaticity coordinates of blue light micro-RCLEDs under varying excitation power. Without CF, as the current gradually increases, the blue light micro-RCLED experiences a Quantum Confined Stark Effect (QCSE), leading to a blue shift in wavelength [25, 29]. The leaked blue light affects the overall color purity, causing a shift in CIE coordinates, as shown in Fig. 4a. On the contrary, after the addition of a yellow CF, the blue light leakage, which blue shifts due to the QCSE phenomenon, can be effectively suppressed. Thus, the chromaticity coordinates can almost remain constant, as depicted in Fig. 4b. Notably, after adding a yellow CF, the color purity of RGY QDPR significantly increases, with RGY improving by 14.7%, 21.5%, and 15.3%, respectively. Such results demonstrate extremely high wavelength stability, beneficial for the development of high-end display products. Moreover, the study also compared the wavelength shift of commercial micro-LEDs under varying driving currents for RGB, as shown in Fig. 4c. From the figure, it is observed that commercial micro-LEDs are severely affected by QCSE, exhibiting significant wavelength shifts, with a substantial blue shift at high driving currents. This is highly disadvantageous for applications, making the use of QDPR CCL a crucial step in driving the development of future display technologies.

**Fig. 5:**
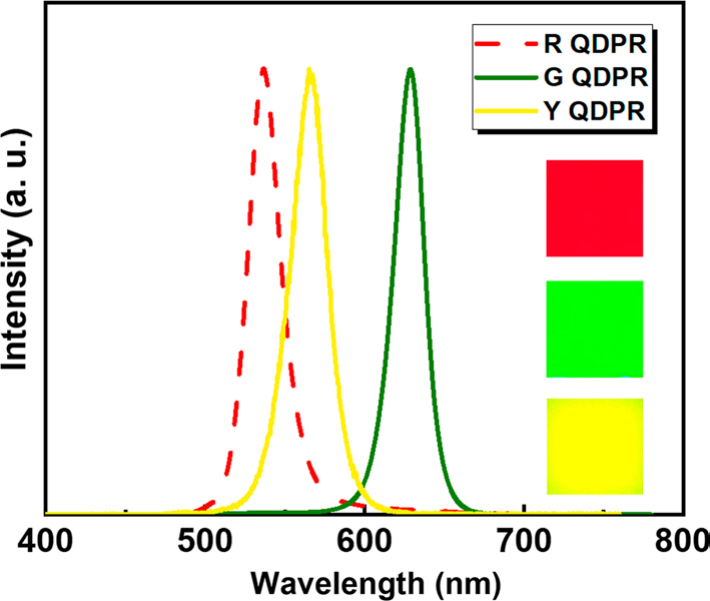
RGY QDPR + Yellow CF CCL measurement emission spectra

Following previous research that determined the selection of yellow pigment for the CF, QDPR CCL were fabricated for further study and analysis. Figure 5 shows the emission spectrum of RGY QDPR + YCF CCL. Optical properties were measured using a spectrometer (Maya 2000 Pro), with the wavelengths of RGY QDPR + YCF CCL being 629 nm, 537 nm, and 565.3 nm, respectively. The Photoluminescence Quantum Yield (PLQY) was measured using a commercial device developed by Otsuka Tech Electronics Co., Ltd. Electronics (TQ-10), with the PLQYs of RGY QDPR + YCF CCL being 78.0%, 66.6%, and 73.5%, respectively. Due to the self-aggregation effect of QDs, the uniformity of illumination in yellow light photolithography processes for QDs is usually poor [13]. By adding nano-scattering particles into the QD ink, the light scattering effect is amplified, increasing the possibility of blue light stimulating the QDs. Moreover, the addition of TiO_2_ can maintain the positioning of QDs to prevent the self-aggregation effect after the spin-coating process, thus enhancing color performance. To analyze the color performance of the emitted light images, the fabricated RGY QDPR CCL was measured using a Fluorescence Optical Microscope (FLOM). The FLOM images were converted to grayscale and divided into 300 × 300 pixels using MATLAB. The uniformity of illumination is defined as the ratio between the minimum value and the average value of the illumination pixels. The illumination uniformity of RGY QDPR was 98.6%, 96.7%, and 73.8%, respectively. As a result, the self-aggregation phenomenon of QDs was effectively suppressed, approaching high color purity, which is very promising for display technology.

**Fig. 6:**
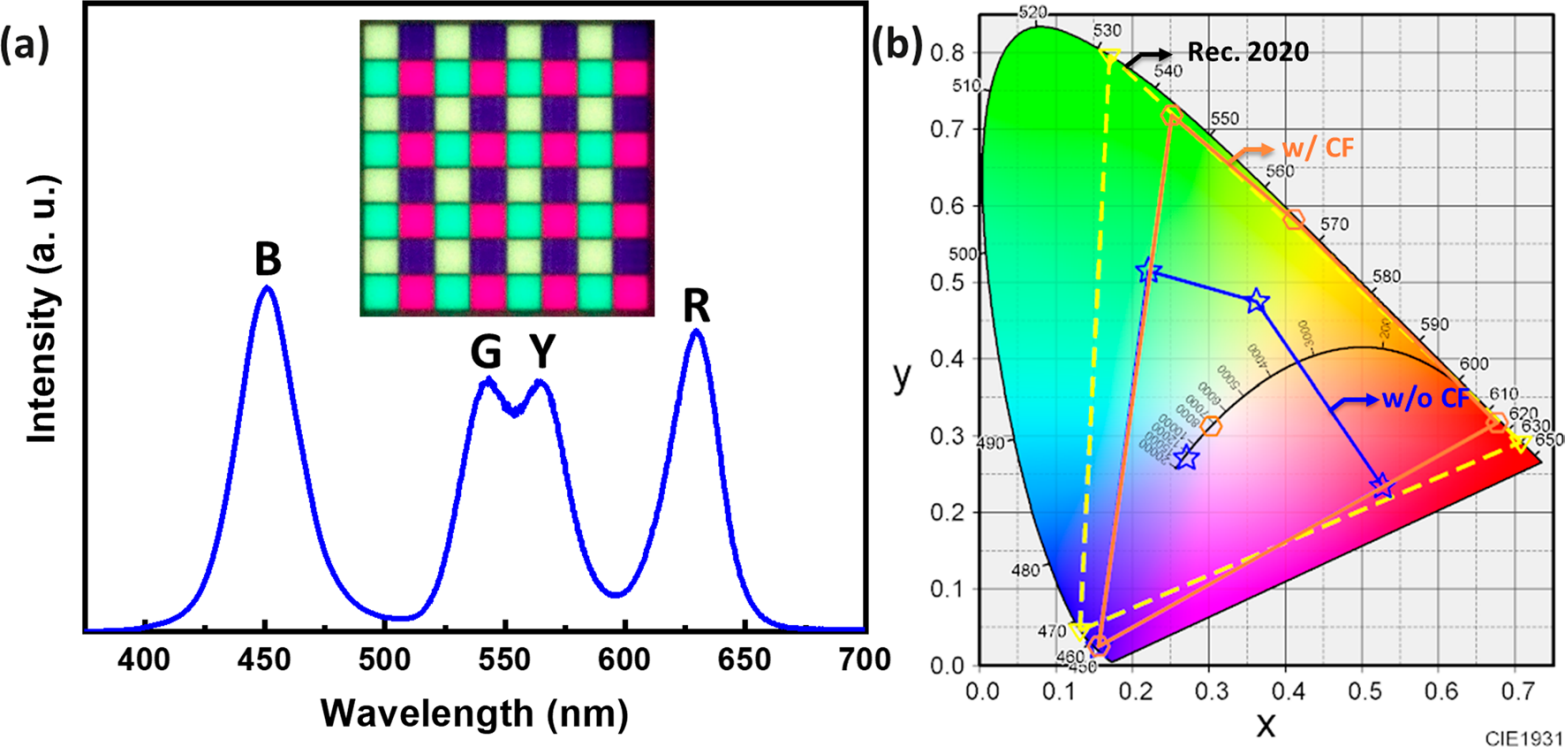
**a** Emission spectra of full-color micro-RCLEDs **b** The CIE-1931 chromaticity diagram of RGBY pixel micro-RCLED

The foundational research previously conducted led to the fabrication of a full-color micro-RCLED array with individual pixel dimensions of 5 × 5 μm^2^ and a spacing of 1 μm, resulting in an 8 × 8 array. Figure 6a displays the spectral figure of the full-color micro-RCLED at a driving power of 2.5 mW (current density = 65 A/cm^2^). Figure 6b examines the impact of adding a yellow CF on the color gamut area. Within the CIE-1931 chromaticity diagram, comparing the national television standards committee (NTSC) and Rec. 2020 chromaticity space standards, the color gamut areas without CF were 55.9% and 41.8%, respectively. With the addition of the yellow CF, the color gamut areas increased to 106.08% and 79.20%, respectively. This enhancement shows that adding a yellow CF brings the color gamut area closer to the Rec.2020 standard due to the yellow pixels' ability to accurately render specific colors, particularly in terms of brightness and color temperature. Such an improvement is crucial for displays seeking a high color gamut as it helps reduce the energy consumption needed to achieve the same visual effects. Furthermore, incorporating yellow sub-pixels allows for easier regulation of white light. Compared to merely mixing red and green light to produce yellow, using yellow pixels provides a brighter yellow without increasing power consumption. This can enhance the energy efficiency of the overall system, especially for displaying brighter images or videos. Additionally, it's noteworthy that a layer of Al_2_O_3_ was applied on top of the QDPR CCL array using low-temperature ALD technology. Past research has proven the effectiveness of using ALD passivation protection technology to coat QD surfaces with oxides [13, 14]. This method effectively protects QDs from any changes due to high temperatures and material characteristics, as well as prevents moisture and oxidation. The developed full-color micro-RCLED significantly improved light leakage and utilized RGBY four-color pixels, showcasing a full-color display with low energy consumption, high color purity, and a wide color gamut area [3, 5, 9, 24].

## Conclusions

In summary, we investigated the optical characteristics of a full-color micro RCLED array using an RGY QDPR array paired with blue light micro-RCLEDs. The additional TiO_2_ scattering particles inside the QDPR ink can increase the path length of incident light and the chances of exciting QDs, while also mitigating the issue of quantum dot self-aggregation. Furthermore, the introduction of an ultra-thin yellow CF further suppressed blue light leakage without significantly reducing the emission intensity of RGY. Additionally, the illumination uniformity of RGY subpixels reached 98.6%, 96.7%, and 73.8%, respectively, achieving excellent color performance. The full-color micro RCLED with RGY QDPR + YCF displayed a color gamut of 106.08% in the NTSC space and 79.20% in the Rec. 2020 standard, realizing a broader color gamut. The combination of RGY QDPR with blue light micro-RCLEDs in a full-color micro-RCLED array, featuring high uniformity and efficient color conversion, offers a display with low energy consumption, high color purity, and a wide color gamut area. This has a broad application prospect for the future of the display industry.

## Data Availability

The data presented in this study are available from the corresponding author upon reasonable request.

## References

[1] Huang J, Hu Z, Gao X, Xu Y, Wang L (2021). Unidirectional-emitting GaN-based micro-LED for 3D display. Opt Lett.

[2] Huang J, Tang M, Zhou B, Liu Z, Yi X, Wang J, Li J, Pan A, Wang L. GaN-based resonant cavity micro-LEDs for AR application. Appl Phys Lett. 2022; 121.

[3] Wilmsen CW, Temkin H, Coldren LA (2001). Vertical-cavity surface-emitting lasers: design, fabrication, characterization, and applications.

[4] Wirth R, Karnutsch C, Kugler S, Streubel K (2001). High-efficiency resonant-cavity LEDs emitting at 650 nm. IEEE Photonics Technol Lett.

[5] Cai W, Yuan J, Ni S, Shi Z, Zhou W, Liu Y, Wang Y, Amano H (2019). GaN-on-Si resonant-cavity light-emitting diode incorporating top and bottom dielectric distributed Bragg reflectors. Appl Phys Express.

[6] Liu Z, Ren K, Dai G, Zhang J (2023). A review on micro-LED display integrating metasurface structures. Micromachines.

[7] Zhang X, Qi L, Chong WC, Li P, Tang CW, Lau KM (2021). Active matrix monolithic micro-LED full-color micro-display. J Soc Inform Display.

[8] Wu Y, Ma J, Su P, Zhang L, Xia B (2020). Full-color realization of micro-LED displays. Nanomaterials.

[9] Kishino K, Sakakibara N, Narita K, Oto T (2020). Two-dimensional multicolor (RGBY) integrated nanocolumn micro-LEDs as a fundamental technology of micro-LED display. Appl Phys Express.

[10] Lei Z, Yu X, Strobel M (2020). High-dynamic-range and wide color gamut video. NANO-CHIPS 2030: on-chip AI for an efficient data-driven world.

[11] Xuening L, Changpo J, Xiaoke L, Zhihao L, Zhengfei Z. Rgbw Led mixing temperature compensation method with high output consistency. Available at SSRN 4551169.

[12] Lee G-Y, Weng S-Y, Ho W-H, Huang C-W, Chao H-Y, Huang S-K, Kuo H-C, Wu C-C, Lin C-C (2023). Photonic characterization and modeling of highly efficient color conversion layers with external reflectors. IEEE Photonics J.

[13] Lee T-Y, Miao W-C, Hung Y-Y, Bai Y-H, Chen P-T, Huang W-T, Chen K-A, Lin C-C, Chen F-C, Hong Y-H (2023). Ameliorating uniformity and color conversion efficiency in quantum dot-based micro-LED displays through Blue–UV hybrid structures. Nanomaterials.

[14] Lee T-Y, Hsieh T-H, Miao W-C, James Singh K, Li Y, Tu C-C, Chen F-C, Lu W-C, Kuo H-C (2022). High-reliability perovskite quantum dots using atomic layer deposition passivation for novel photonic applications. Nanomaterials.

[15] Xuan T, Shi S, Wang L, Kuo H-C, Xie R-J (2020). Inkjet-printed quantum dot color conversion films for high-resolution and full-color micro light-emitting diode displays. J Phys Chem Lett.

[16] Chen S-WH, Huang Y-M, Singh KJ, Hsu Y-C, Liou F-J, Song J, Choi J, Lee P-T, Lin C-C, Chen Z (2020). Full-color micro-LED display with high color stability using semipolar (20–21) InGaN LEDs and quantum-dot photoresist. Photonics Res.

[17] Yin Y, Hu Z, Ali MU, Duan M, Gao L, Liu M, Peng W, Geng J, Pan S, Wu Y (2020). Full-color micro-LED display with CsPbBr 3 perovskite and CdSe quantum dots as color conversion layers. Adv Mater Technol.

[18] Huang Y-M, Chen J-H, Liou Y-H, James Singh K, Tsai W-C, Han J, Lin C-J, Kao T-S, Lin C-C, Chen S-C (2021). High-uniform and high-efficient color conversion nanoporous GaN-based micro-LED display with embedded quantum dots. Nanomaterials.

[19] Kang J-H, Li B, Zhao T, Johar MA, Lin C-C, Fang Y-H, Kuo W-H, Liang K-L, Hu S, Ryu S-W (2020). RGB arrays for micro-light-emitting diode applications using nanoporous GaN embedded with quantum dots. ACS Appl Mater Interfaces.

[20] Song J, Kang J-H, Han J (2021). Monolithic RGB micro-light-emitting diodes fabricated with quantum dots embedded inside nanoporous GaN. ACS Appl Electron Mater.

[21] Lin C-C, Liang K-L, Chao H-Y, Wu C-I, Lin SF, Huang B-M, Huang C-W, Wu C-C, Kuo W-H, Fang Y-H. Fabricating Quantum Dot Color Conversion Layers for Micro-LED-Based Augmented Reality Displays. ACS Appl Opt Mater. 2023.

[22] Hyun B-R, Sher C-W, Chang Y-W, Lin Y, Liu Z, Kuo H-C (2021). Dual role of quantum dots as color conversion layer and suppression of input light for full-color micro-LED displays. J Phys Chem Lett.

[23] Li Z-T, Li J-X, Li J-S, Deng Z-H, Deng Y-H, Tang Y (2020). Scattering effect on optical performance of quantum dot white light-emitting diodes incorporating SiO_2_ nanoparticles. IEEE J Quantum Electron.

[24] Chen C-J, Chen K-A, Kuo W-H, Wu C-I, Shen J-W, Kuo H-C, Chiang R-K. P‐65: strategic realization of the highest possible pixel density for quantum dot color converter with optimized performance. In: Proceedings of SID Symposium Digest of Technical Papers; pp. 1725–1727.

[25] Huang W-T, Lee T-Y, Bai Y-H, Wang H-C, Hung Y-Y, Hong K-B, Chen F-C, Lin C-F, Chang S-W, Han J (2024). InGaN-based blue resonant cavity micro-LEDs with staggered multiple quantum wells enabling full-color and low-crosstalk micro-LED displays. Next Nanotechnol.

[26] Chen K-J, Han H-V, Chen H-C, Lin C-C, Chien S-H, Huang C-C, Chen T-M, Shih M-H, Kuo H-C (2014). White light emitting diodes with enhanced CCT uniformity and luminous flux using ZrO_2_ nanoparticles. Nanoscale.

[27] Chen H-C, Chen K-J, Lin C-C, Wang C-H, Han H-V, Tsai H-H, Kuo H-T, Chien S-H, Shih M-H, Kuo H-C (2012). Improvement in uniformity of emission by ZrO_2_ nano-particles for white LEDs. Nanotechnology.

[28] Bai Z, Hao L, Zhang Z, Huang Z, Qin S (2017). Enhanced photoluminescence of corrugated Al_2_O_3_ film assisted by colloidal CdSe quantum dots. Nanotechnology.

[29] Wu H, Li H, Kuo S-Y, Chen B-Y, Lu T-C, Huang H (2020). High output power GaN-based green resonant-cavity light-emitting diodes with trapezoidal quantum wells. IEEE Trans Electron Devices.

